# 50 Years of Cetacean Strandings Reveal a Concerning Rise in Chilean Patagonia

**DOI:** 10.1038/s41598-020-66484-x

**Published:** 2020-06-11

**Authors:** Mario Alvarado-Rybak, Frederick Toro, Joaquín Escobar-Dodero, Amy C. Kinsley, Maximiliano A. Sepúlveda, Juan Capella, Claudio Azat, Galaxia Cortés-Hinojosa, Natalia Zimin-Veselkoff, Fernando O. Mardones

**Affiliations:** 1 PhD Program in Conservation Medicine, Faculty of Life Sciences, Universidad Andres Bello, Republica 252, Santiago, Chile; 2 Sustainability Research Center (CIS), Faculty of Life Sciences, Universidad Andres Bello, Republica 252, Santiago, Chile; 3 Escuela de Medicina Veterinaria, Universidad Santo Tomás, Av. Limonares 190, Viña del Mar, Chile; 4 Department of Veterinary Population Medicine, College of Veterinary Medicine, University of Minnesota, Saint Paul, MN, USA; 5 Chilean Patagonia Project, The Pew Charitable Trusts, Av. Costanera Andrés Bello 2233, Providencia, Santiago, Chile; 6 Whalesound Ltda, Lautaro Navarro, 1163 P2 Punta Arenas, Chile; 7 Department of Small Animal Clinical Sciences, College of Veterinary Medicine, Gainesville, University of Florida, Florida, 32610 USA; 8 School of Veterinary Medicine, Pontifical Catholic University of Chile, Marcoleta 391, Santiago, 8330024 Chile

**Keywords:** Ecological epidemiology, Ecological modelling, Marine biology

## Abstract

Cetacean strandings (CS) have been reported in increasing numbers in coastal areas worldwide. Although the causes of these strandings are unknown, a number of anthropogenic and environmental factors have been suggested. This paper aims to characterize CS patterns and describe their fine-scale spatiotemporal dynamics. We analysed spatial and spatiotemporal CS patterns in Chile from January 1968 to January 2020. We identified a total of 389 CS events affecting eight cetacean families, 21 genera, and 35 species, which represent more than 85% of the reported species richness for the country. Most CS events (94.1%) were single (*i.e*., ≤two individuals). There were also 18 mass stranding (three to 24 individuals, 4.1%) and nine unusually large mass stranding events (>25 individuals, 2%). Purely spatial tests showed CS events appearing in random occurrence along the Chilean coast. Local tests for spatio-temporal clusters, however, identified a greater number of hotspots reported in the southernmost part of the country, namely, Chilean Patagonia. Specifically, significant spatio-temporal clusters were identified and defined as containing three or more individuals within a two-month period as a focal coastal event (<1 km radius). It is a cause of concern that CS events in Chile have been increasing consistently over the last decades, and although we were not able to identify their causes, we are able to highlight the importance of changes in climate conditions and of an increase in monitoring activities as primary drivers for such patterns, particularly important in Chilean Patagonia.

## Introduction

Marine mammals are prime sentinel species for ecosystems and human health alike^1^. Many of them have long life spans, are long-term coastal residents, feed at a high trophic level, and have unique fat stores that can serve as depots for anthropogenic toxins^1^. Consequently, the study of stranded marine mammals provides valuable records of circulating pathogens and contaminants that could be a risk for coastal populations and provide important information in regard to marine mammals’ biodiversity in coastal areas worldwide^2–4^. Likewise, stranding records and associated attributes^5^ can provide vital information on species richness and diversity by identifying spatial locations and periods of occurrence^6^, and they can also provide a very effective early warning system for the protection of human health^1^.

Cetacean strandings (CS) constitute a worldwide phenomenon, yet the cause of these events remains largely unknown^7^. Although few primary causes have been proposed, there is a general agreement that CS are multifactorial in nature and species dependent^6^. For instance, proposed causes include navigational errors from bathymetric features, coastal configuration; or geomagnetic topography^8,9^; climate or oceanographic events^10,11^; anthropogenic noise and sonar interference^12,13^; pollution^14,15^; infectious diseases^16–18^, and behavioural patterns^19^.

Chile has a high diversity of cetaceans. About 40% of the world’s cetaceans inhabit in Chilean waters^20^, and its coast cover more than 50% of the latitude of the southern hemisphere with more than 8,000 km of coastline. Despite this, there are few studies on strandings or unusual mortality events of cetaceans off the coast of Chile, with most of the stranding records being purely anecdotal. Most studies in Chile focus on determining the aetiology of such strandings^21–25^, but no report has investigated their long term spatial and temporal patterns.

For the present study, we analysed the spatial and spatiotemporal characteristics of 50 years of Chilean cetacean stranding data. These analyses are essential to understand which species are primarily affected and to assess both the spatial extent of CS and its association with temporal patterns of the phenomenon. The results from the study will provide new information for the enhancement management strategies, data requirements, and sampling efforts for future CS events in order to improve conservation policies and contribute to a greater knowledge of the marine and coastal ecosystems in Chile, and possibly even on a global scale.

## Results

### Cetacean stranding events

**Figure 1:**
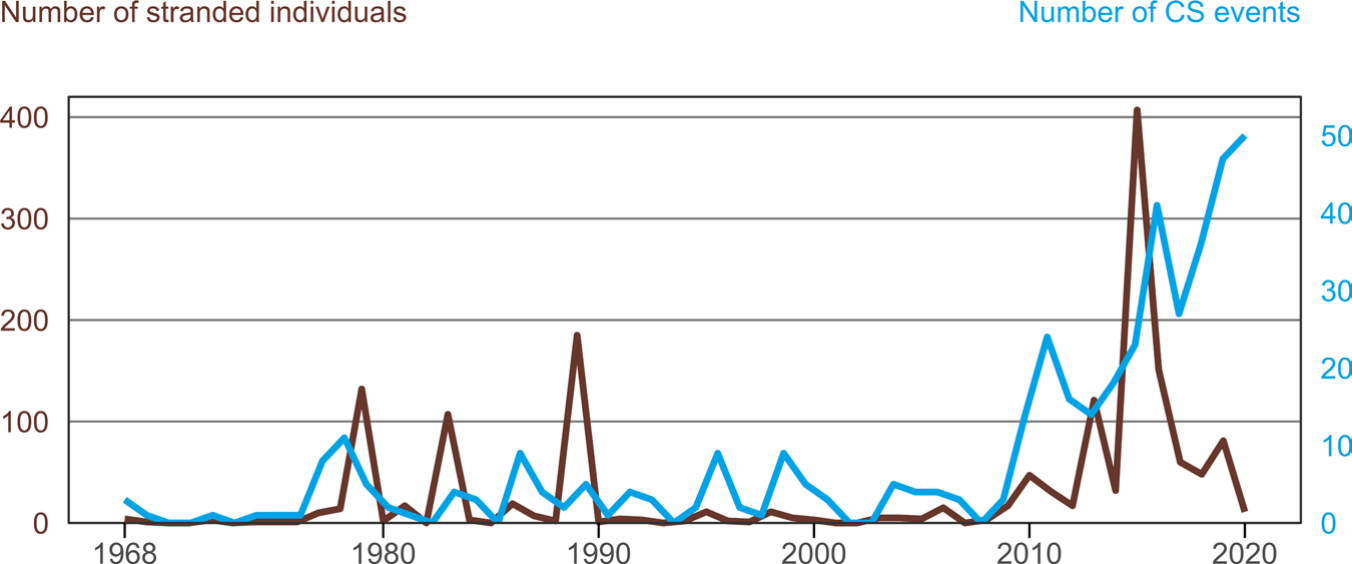
Observed number of stranded cetacean individuals (brown) and number of stranding events (blue line) from January 1968 to January 2020 in Chile.

**Figure 2:**
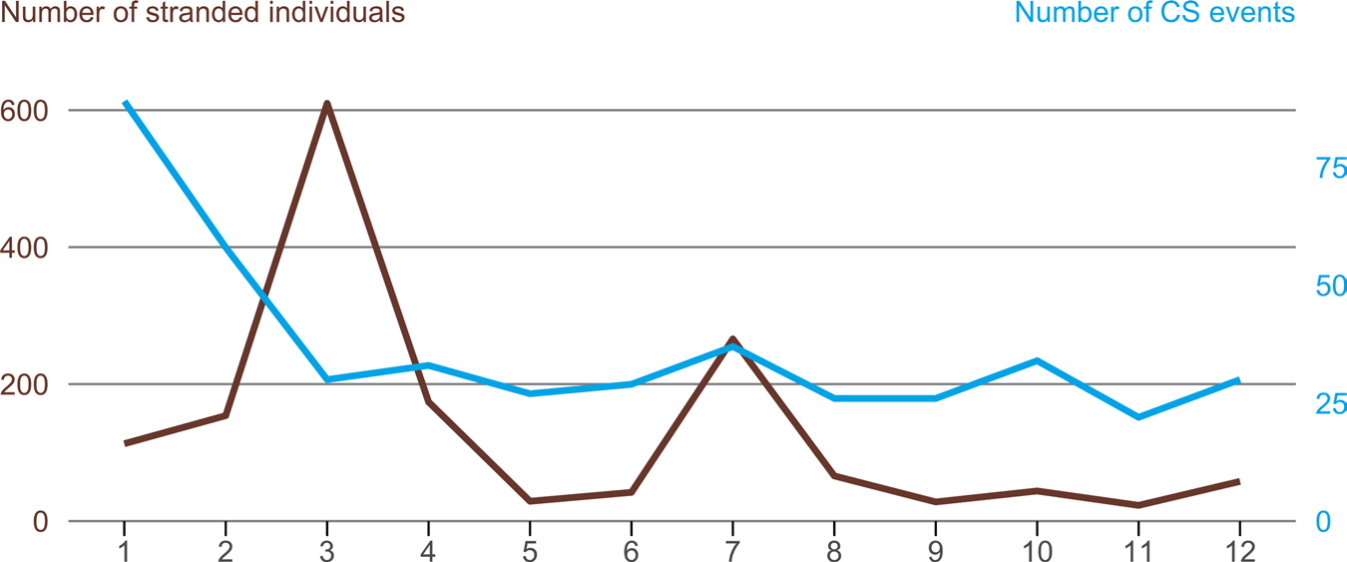
Aggregated sum of all recorded stranded individuals and CS events within-years in Chile from January 1968 to January 2020. Months 1 through 12 refer to January (1) through December (12).

Between January 1968 and January 2020, a total of 441 CS events, affecting 1,607 stranded cetaceans, were recorded along the Chilean coast (Fig. 1). Most CS events (94.1%) were single (*i.e*., ≤two individuals). There were also 18 mass strandings (three to 24 individuals, 4.1%) and nine unusually large mass stranding event (>25 individuals, 2%). Cetacean strandings were reported every month; with 20.2% (n = 89) of total events occurring in January, 13.2% (n = 58) in February, and 8.4% (n = 37) in July. (Fig. 2). Spatially, at least one CS event was reported in 15 out of the 16 administrative regions of Chile. When the locations were aggregated by month, it became apparent that most events occurred in the regions of Valparaíso with 17% (n = 75), Magallanes with 14.5% (n = 64), and Coquimbo 11.3% (n = 50). On the opposite end, the region of Araucanía had the least number of reported events with only two strandings (0.5% of all events). The greatest number of CS events was observed in 2019 with 11.3% (n = 50), followed by the year 2018 with 10.7% (n = 47) and 2015 with 9.3% (n = 41). Regarding the numbers of stranded individuals, the greatest numbers of total stranded cetaceans were reported in March with 37.9% (n = 610), followed by July with 16.5% (n = 266) and April with 10.8% (n = 174) (Fig. 2). The Aysén and Magallanes regions accounted for most of the geographical distribution, with a 33.8% (n = 543) and a 31.5% (n = 506) of the total number of stranded individuals, followed by the Coquimbo region with 9.6% (n = 154). The year 2015 also accounted for the highest number of stranding individuals with 25.3% (n = 407) of the total, followed by the years 1989 and 2016 with 11.1% (n = 185) and 9.4% (n = 151) specimens. Overall, the median of stranded cetaceans in an stranding event was 2 with an interquartile range of 3; and the largest number of stranded cetaceans for a given event was 367 Sei whales that were reported at the Golfo de Penas area in the Magallanes region in March 2015^24^.

### Cetacean species

Cetacean stranding events were reported in eight cetacean families including 21 genera and 35 species. *Odontoceti* and *Mysticeti* species accounted for 74.8% (n = 330) and 25.2% (n = 111) of CS events, respectively; while odontocetes accounted for 66.8% (n = 1,073) and mysticetes for 33.2% (n = 534) of stranded cetaceans. Within stranded odontocetes, most events belonged to the *Delphinidae* 36.3% (n = 160), followed by the *Phocoenidae* 15.4% (n = 68) and *Physiteridae* 10.7% (n = 47) families. *Delphinidae* had the highest number of stranded individuals (n = 865), 80.6% of the *Odontoceti* order and 53.8% of all cetaceans. In the *Mysticeti* suborder, individuals of the *Balaenopteridae* family were the most frequently stranded with 96.3% (n = 514) of cases, followed by members of the *Balaenidae* family at 3.4% (n = 18). In term of stranding events, 87.4% (n = 97) were composed of *Balaenopterids*. If all events are considered together, the *Delphinidae* and *Balaenopteridae* families account for 58.3% (n = 257) of all stranding events, and for up to 85.8% (n = 1,379) of all cetaceans stranded through the years.

At the species level (Table 1), the Burmeister’s porpoise (*Phocoena spinipinnis)* and the sperm whale (*Physeter macrocephalus*) were stranded most frequently, with 66 events and 47 events, respectively. Of these species, sperm whales had a maximum of 17 individuals stranded in a single event, while the Burmeister’s porpoise had only one event with two individuals. The species with the least events were the Gray’s beaked whale (*Mesoplodon grayi*, n = 1), the spade-toothed whale (*Mesoplodon traversii*, n = 1), the spinner dolphin (*Stenella longirostris*, n = 1), Bryde’s whale (*Balaenoptera brydei*, n = 2), the rough-toothed dolphin (*Steno bredanensis*, n = 2), the pygmy right whale (*Caperea marginata*, n = 2), the striped dolphin (*Stenella coeruleoalba*, n = 2), the pygmy beaked whale (*Mesoplodon peruvianus*, n = 2), and the spectacled porpoise (*Phocoena dioptrica*, n = 2). As for the number of stranded individuals per event, there were 367 stranded Sei whale (*Balaenoptera borealis*) individuals reported in one single event at Golfo de Penas in 2015, followed by the false killer whale (*Pseudorca crassidens*) with 181 individuals stranded in a single event in 1989. Both species also account for the most cases of stranded cetaceans across the years with 414 and 337 individuals respectively, followed by the long-finned pilot whale (*Globicephala melas*) with 315 cases. Only six odontocetes were classified as undetermined due to their advanced state of decomposition.

**Table 1:** Stranded cetaceans at Chilean coast between 1968 and 2020.

Common name	Scientific name	Family	N° Individuals	N° Events	IUCN
Southern Right Whale	*Eubalaena australis*	Balaenidae	18	12	LC
Minke Whale	*Balaenoptera acutorostrata*	Balaenopteridae	34	17	LC
Sei Whale	*Balaenoptera borealis*	Balaenopteridae	414	17	EN
Bryde’s Whale	*Balaenoptera brydei*	Balaenopteridae	2	2	LC
Blue Whale	*Balaenoptera musculus*	Balaenopteridae	12	12	EN
Fin Whale	*Balaenoptera physalus*	Balaenopteridae	27	27	EN
Humpback Whale	*Megaptera novaeangliae*	Balaenopteridae	25	22	LC
Pygmy Right Whale	*Caperea marginata*	Cetotheriidae	2	2	DD
Commerson’s Dolphin	*Cephalorhynchus commersonii*	Delphinidae	7	6	LC
Chilean Dolphin	*Cephalorhynchus eutropia*	Delphinidae	14	13	NT
Short-beaked Common Dolphin	*Delphinus delphis*	Delphinidae	15	15	LC
Short-finned Whale	*Globicephala macrorhynchus*	Delphinidae	16	10	DD
Long-finned Whale	*Globicephala melas*	Delphinidae	315	13	DD
Risso’s Dolphin	*Grampus griseus*	Delphinidae	79	15	LC
Peale’s Dolphin	*Lagenorhynchus australis*	Delphinidae	15	15	DD
Dusky Dolphin	*Lagenorhynchus obscurus*	Delphinidae	22	21	DD
Southern Right Whale Dolphin	*Lissodelphis peronii*	Delphinidae	19	18	DD
Killer Whale	*Orcinus orca*	Delphinidae	4	4	DD
False Killer Whale	*Pseudorca crassidens*	Delphinidae	337	10	DD
Striped Dolphin	*Stenella coeruleoalba*	Delphinidae	2	2	LC
Spinner dolphin	*Stenella longirostris*	Delphinidae	1	1	LC
Rough-toothed Dolphin	*Steno bredanensis*	Delphinidae	2	2	LC
Common Bottlenose Dolphin	*Tursiops truncatus*	Delphinidae	17	15	LC
Pygmy Sperm Whale	*Kogia breviceps*	Kogidae	12	10	DD
Dwarf Sperm Whale	*Kogia sima*	Kogidae	15	15	DD
Spectacled Porpoise	*Phocoena dioptrica*	Phocoenidae	2	2	DD
Burmeister’s porpoise	*Phocoena spinipinnis*	Phocoenidae	70	66	DD
Sperm Whale	*Physeter macrocephalus*	Physiteridae	72	47	VU
Arnoux’s Beaked Whale	*Berardius arnuxii*	Ziphiidae	8	5	DD
Blainville’s Beaked Whale	*Mesoplodon densirostris*	Ziphiidae	3	3	DD
Gray’s beaked whale	*Mesoplodon grayi*	Ziphiidae	1	1	DD
Layard’s Beaked Whale	*Mesoplodon layardii*	Ziphiidae	5	3	DD
Pygmy Beaked Whale	*Mesoplodon peruvianus*	Ziphiidae	3	2	DD
Spade-toothed Whale	*Mesoplodon traversii*	Ziphiidae	1	1	DD
Cuvier’s Beaked Whale	*Ziphius cavirostris*	Ziphiidae	10	9	LC

IUCN Redlist of threatened species criteria by species are Data Deficient (DD), Least Concern (LC), Vulnerable (VU) and Endangered (EN).

### Time series analysis

Observed CS events were scattered throughout the time series with several peaks for some years, which were highest during the latest decade (Fig. 1). The Augmented Dickey–Fuller test was non-significant (*p* = 0.3) so the time series is considered as non-stationary with an increasing trend over time. The estimated trend component shows that the number of CS events appear to increase over time (Seasonal Mann-Kendall trend test *p* < 0.01); however, the probable changing point in time for the trend was identified at September 2008 (Pettitt’s test *p* < 0.01). Decomposition function identified seasonal variability in the number of CS events per month, indicating that there is a peak every summer and every winter, a pattern that is repeated every year (Fig. 2). The estimated seasonal factors were consistent throughout the years with the largest seasonal factors for January (0.84, summer), February (0.45, summer), and July (0.01, winter), and the lowest were November (−0.28) and September (−0.21). These results suggest that CS peak considerably during summer and, in a lesser extent, in winter and decline from September to November each year. The autocorrelation function (ACF) and partial ACF (PACF) showed no significant correlation or lag in the stranding data.

### Spatial and space-time cluster analyses

**Figure 3:**
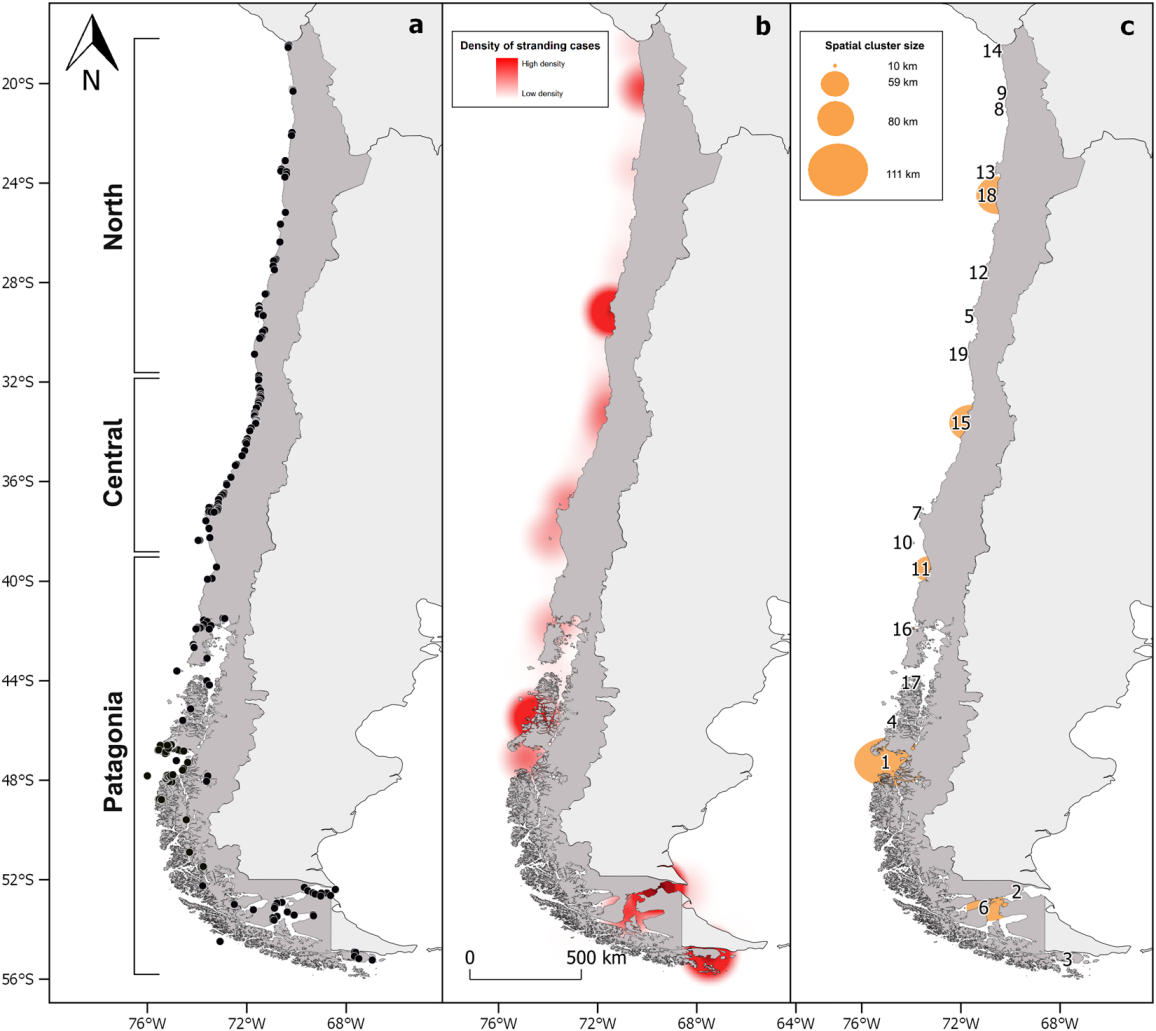
(**a**) Stranding events (black dots) along Chile since 1968 to 2020. (**b**) Heatmap that illustrates density of stranding cases along Chile. (**c**) Distribution of 19 statistically significant (*p* < 0.001) cetacean stranding clusters along the Chilean coast as described by the permutation space–time analyses (see Table 1).

From January 1968 to January 2020, CS events were reported in 680 locations throughout the Chilean coast (Fig. 3a). The density of the CS events depicted in Fig. 3b highlights four zones that appear to have higher densities of CS events. Three of these four zones were at the southernmost part of Chile and one was at the northcentral part of the country (approximately 72°W–28°S). The Global Moran’s *I* index was statistically insignificant (*p* = 0.79).

The permutation space-time analyses identified a total of 19 statistically significant spatiotemporal clusters (*p* < 0.01) as described in Table 2 and shown in Fig. 3c. About 68% of these clusters (13/19) were CS events located in a single location (<1 km), referred to as statistically significant punctual or focal CS events, and ranged from three to 181 stranded cetaceans in each cluster. The remaining clusters (n = 6) were of a variable radius, ranging from ten to 111 km (mean = 67 km and median = 70 km) with four to 353 stranded cetaceans.

**Table 2:** Statistically significant clusters (p < 0.001) detected by spatiotemporal permutation model using the space–time scan statistic for the cetacean strandings between January 1968 and August 2018, in Chile. Obs.: observed; Exp.: expected.

Cluster	Region	Zone	Latitude	Longitude	Radius (km)	Time frame	Number of cases	Exp. cases	Obs./Exp.	*p*-value
1	Aysén	Patagonia	47.21 S	74.83 W	111	2015/3/1 to 2015/3/31	353	88.9	3.9	<0.001
2	Magallanes	Patagonia	52.45 S	69.54 W	0	1989/3/1 to 1989/3/31	181	20.4	8.9	<0.001
3	Magallanes	Patagonia	55.18 S	67.49 W	0	1979/4/1 to 1979/4/30	125	9.72	12.9	<0.001
4	Aysén	Patagonia	45.6 S	74.58 W	0	2016/7/1 to 2016/7/31	124	9.72	12.8	<0.001
5	Coquimbo	North	29.25 S	71.46 W	0	1983/7/1 to 1983/7/31	103	7.2	14.4	<0.001
6	Magallanes	Patagonia	53.08 S	70.87 W	60	2013/1/1 to 2013/2/28	92	6.2	14.5	<0.001
7	Bío Bío	Central	37.19 S	73.56 W	0	2017/12/1 to 2017/12/31	25	0.4	61.8	<0.001
8	Tarapacá	North	20.24 S	70.13 W	0	2011/6/1 to 2011/6/30	13	0.1	123.6	<0.001
9	Tarapacá	North	20.24 S	70.13 W	0	2010/4/1 to 2010/4/30	15	0.2	68.5	<0.001
10	Bío Bío	Central	38.35 S	73.95 W	0	2013/8/1 to 2013/8/31	10	0.06	146.1	<0.001
11	Los Ríos	Patagonia	39.43 S	73.21 W	58	2019/8/29 to 2019/11/28	7	0.08	89.3	<0.001
12	Atacama	North	27.48 S	70.88 W	0	1986/12/29 to 1987/1/28	4	0.01	401.8	<0.001
13	Antofagasta	North	23.44 S	70.6 W	0	2014/10/1 to 2014/10/31	4	0.02	229.8	<0.001
14	Arica	North	18.54 S	70.33 W	0	2010/10/1 to 2010/10/31	4	0.02	178.6	<0.001
15	Valparaíso	Central	33.55 S	71.6 W	80	2014/12/1 to 2015/2/28	12	0.6	16.6	<0.001
16	Los Lagos	Patagonia	41.85 S	73.96 W	10	2014/7/1 to 2014/7/31	4	0.03	142.8	<0.001
17	Aysén	Patagonia	44.01 S	73.6 W	0	2012/12/1 to 2012/12/31	3	0.006	535.7	<0.001
18	Antofagasta	North	24.37 S	70.54 W	84	2019/3/29 to 2019/4/28	4	0.04	114.8	<0.001
19	Coquimbo	North	30.77 S	71.70 W	0	2018/11/29 to 2018/12/28	3	0.01	267.8	<0.001

Chronologically, the first spatiotemporal cluster was identified in April 1979 (Cluster 3), followed by one spatiotemporal cluster identified in July (Cluster 5), 1983. Other spatiotemporal cluster were identified by December 1986 (Cluster 12) and March 1989 (Cluster 2). No spatiotemporal clusters were reported between 1990 and 2009. There were 15 spatiotemporal clusters reported between 2010 and 2019 (15/19 or 79%).

Space-time clusters were scattered throughout Chile. The number of clusters at each zone was evenly distributed with eight, three and eight spatiotemporal clusters for the North, Central and Patagonia zones, respectively. During the study period, spatiotemporal clusters with most stranded cetaceans were reported at the Patagonia zone (n = 8). Considering all significant spatiotemporal clusters and averaging their temporal extensions and number of stranded cetaceans, we propose a time lag of (±) 2 months to monitor the event, and a number of stranded individuals greater than 3 to be classified as a mass event (see Table 2).

## Discussion

During the last two decades, reports of CS in Chile have been steadily increasing over time, urging an immediate response to understand the causes of this phenomena. Our dataset was built from official and non-official sources that enabled us to carry out the first comprehensive study that synthesized long-term records of CS in Chile. Overall, there were three critical results from our analyses. First, a large number of spatiotemporal clusters were detected along the Chilean coast, highlighting the need for monitoring and surveillance activities along the entire coast, giving particular relevance to Patagonia. Second, with our data, the occurrence of most clusters was established to take place during the last decade, which may be a reflection of augmented public awareness and reporting, policy changes, or the effect of oceanographic and ecological changes. Third, we provide applications for future stranding investigations by determining the time lag to monitor the event (±2 mo.) and the number of stranded individuals for the CS to be classified as a massive event (greater than 3 stranded individuals). Although most CS events would be restricted to a limited spatial extent (<1 km radius), monitoring should also consider about (±) 70 km radius of coastal extension. We also discuss the within-year variation of reporting, the identification of hot spots in different areas of Chile and the species composition of the dataset. In the last year, there have been 61 CS events, 15 of them (24.6%) occurring between December 2019 and January 2020 (last summer season). All these CS events reported a total of 92 stranded individuals that were extensively distributed in the Chilean coast. However, a single event including 29 individuals of *B. borealis* was reported from Chilean Patagonia, one the significant clusters reported here. This demonstrates how the combination of GIS applications, time series, and spatiotemporal analyses can be used to gain a better understanding for the management of CS events in an extended region.

Cetacean stranding events can provide insight into the distribution and population aspects for some species^26^. Here, it was expected that the number of CS events and stranded individuals would mirror the species richness and relative abundance in Chile^27^. In fact, from nine existent families, 24 genera and 41 species that have been reported in Chilean waters^26^, CS events reported here identified 88.9% of cetacean families, 87.5% and 85.4% of genera and species, respectively. In this context, live-dead CS metrics from different countries indicated that strandings showed greater species richness than live surveys and that species richness also increased with coastline length^27^. This was observable for most species, but it was unusually high for *P. spinipinnis*, reported in 43 events. *Phocoena spinipinnis* are a group of porpoises that regularly strand in coastal areas worldwide^3^. It has been described that the leading causes of death are from bycatch and naval presence^28^. In our study, the strandings of *P. spinipinnis* took place for the most part in major ports and fisheries located in central Chile. A different situation occurred with *B. borealis* (n = 387), with 13 reported CS throughout the study period. However, a single event reported about 95% of total reported stranded individuals. This UME occurred in Golfo de Penas and Puerto Natales^24^. The most likely cause of this UME was a toxic algal bloom, but other causes could not be ruled out^24^. In both cases, CS events are useful to understand species distribution and to contrast them with reported richness. Nevertheless, interpretation of these events needs to be taken cautiously.

In this study, it was shown that the distribution of CS events and significant spatiotemporal clusters (Fig. 3) were widely spread along the Chilean coast, indicating that stranding phenomena are relevant throughout the country. However, when looking at the magnitude or number of individuals stranded at each event, more than half of the stranded cetaceans were reported at the southernmost regions of Chile. In other words, these findings suggest that, although the likelihood of a CS event is a random process along the Chilean coast (North and Central zones), a mass stranding or an UME should be expected to take place in the Patagonia zone in the southernmost part of the country (First four significative clusters *p* < 0.001, Table 2). Since CS events have become widespread, implementing a national monitoring strategy would be a logical step, but in terms of an effective rescue and rehabilitation plan for stranded individuals, efforts should be focused in Chilean Patagonia. A feasible example of a risk-based and integrated monitoring plan would be the partnership with the Chilean salmon industry. Marine sites for salmon farming activities are scattered at the southern regions, mostly located at isolated areas and manned by staff and personnel for 24 hours a day for at least 10 months throughout the year. Such productive units can become voluntary monitoring stations and act as an early warning system for either single or multiple strandings. Also, our work provides key indicators (CS frequency, size, duration, extension, etc.) that can be of use for a number of initiatives that might promote management or conservation plans for cetaceans and other marine animals.

The analysis of the within-year variation indicates that although CS events were reported at all times of the year, the months from February to April (summer and early autumn) and July (winter) account for 41% of all CS events. The patterns of late spring and summer strandings have been previously reported for patterns in different species^29–31^, likely due to the seasonality of the animals’ movements and foraging habitat^29,31^. Summertime also favours more visibility because of longer and increased daylight, reduced rainfall, and being coincidental with the “summer holidays”, thus making it more likely for people to detect and report CS events. This potential “observer bias” in reporting is also apparent in July, where, despite low light and poor weather, the “winter holidays” increase the number of observers and reports of CS events. There are additional factors that can also explain the seasonality of CS events reported here, including the intensification of fishing efforts^32^, cetacean breeding activities^33^ or species-specific patterns^34^. Unfortunately, we were not able to accurately classify the source or entity of the reporting for each event (primary reporting entity), *i.e*., naval personnel, fishery inspectors, researchers or visitors. We were also unaware of whether or not this information was collected in the field. The reporting entity is important as it can provide insights about the value of citizen science as a way to contribute to the wealth of information about population structures, distribution, and behaviour, as well as providing assistance with cetacean conservation^35^. We acknowledge the potential for volunteers (‘citizen science’) in environmental monitoring to bring value, both economic and educational, into wildlife research^36^. Our study highlights the importance of the implementation of a systematic collection of CS events, particularly how the entity that reported each event should be specified in order to quantify the value of potential stranding network partners.

From the output of the spatiotemporal analysis (Table 2), it is possible to suggest key indicators that may enhance future monitoring and surveillance activities in Chile. For example, for all significant spatiotemporal clusters (*p* < 0.001), the median extension size (radius) was 27 km and the median time frame (months) was 2 months. These indicators may suggest that whenever a CS occurs, response activities are expected to be in place for about 2 months and to extend over 50 km of coastline. Moreover, it is possible to obtain the median value of the expected number of stranded cetaceans, which may reflect the expected number of stranded cetaceans in a given event. In this study, this indicator ranged from 3 to 10 stranded animals, suggesting that a massive CS should be declared if 3 or more animals are found. Outputs from our spatiotemporal approach provide key indicators for guidance on preparedness and response in an ongoing stranding and also for the evaluation of monitoring activities. Implementing such a technique can be applied in other regions where records of CS are available, including location (geographic coordinates), and number of individuals and species (all data related to CS).

There are few studies in other regions of the world that investigate stranded cetaceans involving periods longer than 25 years. These studies include CS at the Irish coast from 1901 to 1995 (529 events in 94 years)^37^; on Sable Island in Nova Scotia, Canada, (102 events in 28 years) from 1970 to 1998^38^; in Costa Rica (35 events in 33 years) from 1966 to 1999^39^; in the Galapagos Islands (87 events in 80 years) in Ecuador from 1923 to 2003^40^; in the main Hawaiian Islands from 1937 to 2002 (202 events in 65 years)^41^, in Tunisia (132 events in 72 years) from 1937 to 2009^42^; and in South Australia (1,078 events in 127 years) from 1881 to 2008^43^, which is the most extensive, in terms of time, of all assessed studies. Roughly speaking, the proportion of events per year considered in these studies was estimated at 3.95 (*i.e*., average number of events divided by years in the study). In our study, this proportion was estimated at 7.1 events per year, which is 80% higher than the global estimate. The only region to report an even greater proportion was South Australia^43^. We hypothesized that main drivers for this higher reporting of events in Chile would be associated to the country cetacean richness (40% of all cetaceans reported worldwide are present in Chilean waters^37^), the extensive distribution of coastal human populations and activities related to fisheries and aquaculture, and some side effects due to climate change, such as increasing toxic algal bloom in Patagonia^24^, which is linked to changes in water nutrient availability in some areas^44^.

The need to increase the reporting of strandings is evident. As a consequence, it is recommended that the marine mammal network of sightings in Chile should be strengthened by NGOs, Sernapesca and Directemar, and that a national stranding network should be established, integrating the participation of a wide range of actors from civil society and collecting information in a centralized database with the latitude and longitude of the events for further spatial analysis (including GIS tools). The implication of such CS studies may promote the allocation of resources towards more effective monitoring and surveillance of these events in that region. In addition, it is critical to be able to respond quickly and efficiently to these events by bringing together a multidisciplinary team for the investigation and sampling of these events. Future research correlating standings with oceanographic/climatic conditions may help to explain documented patterns, but the effects of increased monitoring efforts need to be accounted for as well.

In conclusion, this study provides insights into the historical patterns of cetacean strandings along the Chilean coast. Reports of CS events have increased alarmingly during the last two decades, particularly in Patagonia. However, it is not clear whether changes in human population, facilities used for reporting, general awareness or climate change would account for variability in reported strandings and explain potential biases. In any case, the use of spatiotemporal analyses provides results that may enhance current monitoring efforts by defining the expected numbers of stranded cetaceans, and the spatial and temporal extension needed after the report of a CS event. The composition of the species in the strandings database reflects the high diversity of cetaceans in Chile, with only ten species known to occur in Chilean waters not recorded in strandings. This is probably associated with more cryptic species with few records in Chile and lack of recognition of some stranded specimens. Our work here provides key indicators (frequency, size, duration, and extension of CS, among others) that can be of use for a number of initiatives that would promote conservation plans for cetacean and other marine animals.

## Methods

### Occurrence data for cetacean strandings in Chile

In Chile, the National Fisheries Service (Sernapesca) and the Maritime Technological Directorate dependent on the Chilean Navy (Directemar) have collected reports on CS occurrences since 1983. Additional occurrences were collected from 1) literature searches conducted in English and Spanish, and from 2) grey literature including proceedings from past conferences, Chilean newspapers, magazines, and local reports. We searched three main electronic databases: Web of Science, PubMed, and the Scientific Library Online (SciELO), using multiple keywords and expressions (strand* OR stranding*) AND (cetacean* OR dolphin* OR porpoise* OR whale*) AND (Chile OR Pacific OR South America). References cited in retrieved reports were reviewed to identify additional reports, which, if not available online, were requested and scanned through the library of the Pontifical Catholic University of Chile. Titles and abstracts were imported into a reference manager system (EndNote, version X7, Thomson Reuters, Carlsbad, CA, USA). Upon the identification of a CS (see above), information was extracted and put into a spreadsheet where each row corresponded to a specific event with the number of individuals involved in the event, date, geographical coordinates of the reported location, and cetacean species. Occurrences were inspected to avoid inaccurate reports and to remove duplicate events. For example, there were a few reports where geographical coordinates referenced places far away from the coastline or located on land, within the country, so these were excluded for the analyses. These activities were conducted independently by two of the co-authors of this study.

### Descriptive and time series analyses

Descriptive analyses were carried out to represent the overall characteristics associated with CS events during the study period from February 1968 to January 2020. Cetacean stranding events for this study were defined as single, including mother and calf, or more strandings during the study period. Furthermore, we defined as mass stranding events (MSE) those when more than three animals stranded and as unusual mortality events (UME) those when the stranded die off in larger numbers than “normal”^45^. To verify the stationarity of the CS data, *i.e*., that the mean and variance of the time series are constant over time, we performed the Augmented Dickey-Fuller test implemented in the ‘tseries’ package^46^ using the statistical software R^47^. We quantified the number of events that occurred monthly and developed an additive time series analysis model. We used an additive model since the random fluctuations in the CS data were roughly constant in size over time. Classical time- series decomposition analysis was performed with the function ‘decompose’ to estimate the seasonal, trend and random components of the CS events using moving averages^48^. Additionally, we performed the non- parametric seasonal Mann-Kendall and Pettitt´s tests to detect seasonally adjusted monotonic trends and single change-point in the time series respectively, implemented in the package ‘trend’^49^. We also explored the autocorrelation and partial autocorrelation functions (ACF and PACF, respectively) to examine the independence of CS events from each other.

### Spatial visualization and space-time analyses

Visualization of CS event locations was achieved using ArcGIS Pro (v2.2.0)^50^ and projected for analysis using WGS 1984 datum as a coordinate system. Spatial distribution of CS events was characterized by the Moran’s *I* test^51^, which is an autocorrelation analysis to identify spatial autocorrelation globally. For the identification of local spatiotemporal autocorrelation analysis, clustering of CS was modelled using the space-time permutation model of the scan statistic test implemented in the SaTScan^TM^ software version 9.4.4^52^. The model was run using only stranding locations and starting dates under the null hypothesis that strandings were randomly distributed in space and time. The model was set to scan for areas with high case numbers so that they test for clusters with a spatial and temporal occurrence that is higher than that outside the cluster. A case was defined as a single stranding event (independently of the number of stranded individuals) that occurred in a single location. Briefly, the number of observed and expected events was counted by a scanning window that moved across space and/or time for each location and variable window size^53^. Among these, the clusters with the greatest difference between observed and expected events were noted. The statistical significance of these clusters were then evaluated considering the multiple testing stemming from the many potential cluster locations and sizes evaluated^54^. The maximum size of the temporal window was set to a three month study period to comprise all four seasons (*i.e*., winter, spring, summer and autumn). The maximum spatial extension of clusters was set to a circular radius of 120 km, based on the estimated size of the spatial extent of the largest *Balaenoptera borealis* strand ever recorded in the world, by March 2015, at Golfo de Penas in Chile^24^.

Distributions of the likelihood ratio and its corresponding *P* value were obtained using the Monte Carlo simulation by generating 999 replications of the data set under the null hypothesis of random distribution of cases in time and space^57^. The test statistics were computed for each random replication, and if the latter was in the most extreme 5% of all test statistics calculated, then the hypothesis test was deemed significant at *p* < 0.05. To interpret and discuss the results, the Chilean coast was split into three distinct geographical zones (North, Central and Patagonia), and the spatiotemporal clusters were assigned to each zone.

## Supplementary information

Dataset 1.

## References

[1] Bossart GD (2011). Marine mammals as sentinel species for oceans and human health. Vet. Pathol..

[2] MacLeod CD (2005). Climate change and the cetacean community of north-west Scotland. Biol. Cons..

[3] Leeney RH (2008). Spatio-temporal analysis of cetacean strandings and by-catch in a UK fisheries hotspot. Biodivers. Conserv..

[4] Pyenson ND (2010). Carcasses on the coast: measuring the ecological fidelity of the cetacean stranding record in eastern North Pacific Ocean. Paleobiology..

[5] Chan, D.K.P., Tsui, H.C.L. & Kot, B.C.W. Database documentation of marine mammal stranding and mortality: current status review and future prospects. *Dis. Aquat. Organ*. **126**, 247–256; 353; 1|0.3354/dao03179 (2017). 10.3354/dao03179 29160222

[6] Perrin, W.F. & Geraci, J.R. Strandings in *Encyclopedia of Marine Mammals* (eds. Perrin, W. F., Würsig, P. B. & Thewissen, J. G. M.). 1192–1194 (Academic Press, 2002).

[7] Cordes DO (1982). The causes of whale strandings. New Zeal. Vet. J..

[8] Kirschvink JL, Dizon AE, Westphal JA (1986). Evidence from strandings for geomagnetic sensitivity in cetaceans. J. Exp. Biol..

[9] Brabyn MW, McLean IG (1992). Oceanography and coastal topography of herd stranding sites for whales in New Zealand. J. Mammal..

[10] Mignucci-Giannoni AA, Toyos-Gonzalez GM, Perez-Padilla J, Rodriguez-Lopez MA, Overing J (2000). Mass stranding of pygmy killer whales (*Feresa attenuata*) in the British Virgin Islands. J. Mar. Biol. Assoc. U.K..

[11] Evans K (2005). Periodic variability in cetacean strandings—links to large-scale climate events. Biol. Lett..

[12] Balcomb KC, Claridge DE (2001). A mass stranding of cetaceans caused by naval sonar in the Bahamas. Bahamas J. Sci..

[13] Madsen PT, Møhl B, Nielsen BK, Wahlberg M (2002). Male sperm whale behavior during exposures to distant seismic survey pulses. Aquat. Mamm..

[14] Alonso MB (2014). Anthropogenic (PBDE) and naturally-produced (MeO-PBDE) brominated compounds in cetaceans - a review. Sci. Total Environ..

[15] Durante CA, Santos-Neto EB, Azevedo A, Crespo EA, Laison-Brito J (2016). POPs in the South Latin America: Bioaccumulation of DDT, PCB, HCB, HCH and Mirex in blubber of common dolphin (*Delphinus delphis)* and Fraser’s dolphin (*Lagenodelphis hosei)* from Argentina. Sci. Total Environ..

[16] Bennett PM, Jepson PD, Law RJ (2001). Exposure to heavy metals and infectious disease mortality in harbour porpoises from England and Wales. Environ. Pollut..

[17] Arbelo M (2013). Pathology and causes of death of stranded cetaceans in the Canary Islands (1999–2005). Dis. Aquat. Organ..

[18] Profeta F (2015). Retrospective seroepidemiological investigations against Morbillivirus, *Toxoplasma gondii* and *Brucella* spp. in cetaceans stranded along the Italian coastline (1998-2014). Res. Vet. Sci..

[19] Odell DK, Asper E, Baucom J, Cornell L (1980). A recurrent mass stranding of false killer whales, *Pseudorca crassidens*, in Florida. Fish Bull..

[20] Viddi F, Hucke-Gaete R, Torres-Florez JP, Ribeiro S (2010). Spatial and seasonal variability in cetacean distribution in the fjords of northern Patagonia, Chile. ICES J. Mar. Sci..

[21] Canto J, Ruiz P, Cárdenas J (1991). Necropsy of southern right whale *Eubalaena australis* and considerations for management of the species. Bol. Mus. Nac. Hist. Nat..

[22] Sanino GP, Hamilton-West C, Rojas A, Yáñez J, Van Waerebeek K (2003). Estudios de restos varados de *Delphinus delphis* y primer registro documentado de pheumonía abscedativa, en Chile. Bol. Mus. Nac. Hist. Nat..

[23] Haro D (2015). Nuevo varamiento masivo de orca falsa, *Pseudorca crassidens*, en el Estrecho de Magallanes, Chile. Rev. Biol. Mar. Oceanogr..

[24] Häussermann V (2017). Largest baleen whale mass mortality during strong El Niño event is likely related to harmful toxic algal Bloom. PeerJ..

[25] Alvarado-Rybak M (2019). A Mass Stranding Event of Long-Finned Pilot Whales. Aquat. Mamm..

[26] Capella, J. & Gibbons, J. Mamíferos marinos in *Biodiversidad de Chile: Patrimonio y* Desafíos. (eds. MMA). 234–244 (Ediciones MMA, 2018).

[27] Pyenson ND (2011). The high fidelity of the cetacean stranding record: insights into measuring diversity by integrating taphonomy and macroecology. Proc. R. Soc. Lond. B Biol. Sci..

[28] Wright AJ, Maar M, Mohn C, Nabe-Nielsen J, Siebert U (2013). Possible Causes of a Harbour Porpoise Mass Stranding in Danish Waters in 2005. PLoS One..

[29] Norman SA (2012). The application of GIS and spatio-temporal analyses to investigations of unusual marine mammal strandings and mortality events. Mar. Mammal Sci..

[30] Delgado A, Ortega J, Sánchez A (1994). Varamientos de mamíferos marinos durante primavera y otoño y su relación con la actividad humana en el norte del Golfo de California. An. Inst. Biol. Univ. Nac. Autón. Méx. Ser. Zool..

[31] Norman SA (2004). Cetacean strandings in Oregon and Washington between 1930 and 2002. J. Cetac. Res. Manage..

[32] Fruet PF (2012). Temporal trends in mortality and effects of by-catch on common bottlenose dolphins, *Tursiops truncatus*, in southern Brazil. J. Mar. Biol. Assoc. U.K..

[33] Prado JH, Mattos PH, Silva KG, Secchi ER (2016). Long-term seasonal and interannual patterns of marine mammal strandings in subtropical western South Atlantic. PLoS One..

[34] Mitchell E (1968). Northeast Pacific stranding distribution and seasonality of Cuvier’s beaked whale *Ziphius cavirostris*. Can. J. Zool..

[35] Hand E (2010). Citizen science: People power. Nature..

[36] Greenwood JJD (1994). Trust the wildlife volunteers. Nature..

[37] Berrow SD, Rogan E (1997). Review of cetaceans stranded on the Irish coast, 1901–95. Mammal Rev..

[38] Lucas ZN, Hooker SK (2000). Cetacean strandings on Sable Island, Nova Scotia, 1970–1998. Can. Field. Nat..

[39] Rodriguez-Fonseca J, Cubero-Pardo P (2001). Cetacean strandings in Costa Rica (1966-1999). Rev. Biol. Trop..

[40] Palacios DM, Salazar SK, Day D (2004). Cetacean remains and strandings in the Galápagos Islands, 1923–2003. LAJAM..

[41] Maldini D, Mazzuca L, Atkinson S (2005). Odontocete Stranding Patterns in the Main Hawaiian Islands (1937–2002): How Do They Compare with Live Animal Surveys?. Pac. Sci..

[42] Karaa S, Bradai MN, Jribi I, El Hili HA, Bouain A (2012). Status of cetaceans in Tunisia through analysis of stranding data from 1937 to 2009. Mammalia..

[43] Segawa T, Kemper C (2015). Cetacean strandings in South Australia (1881-2008). Aust. Mammal..

[44] Hoegh-Guldberg O, Bruno JF (2010). The impact of climate change on the World’s marine ecosystems. Science..

[45] Groom C, Coughran D (2012). Three decades of cetacean strandings in Western Australia: 1981 to 2010. J. R. Soc. West. Aust..

[46] Trapletti, A. & Hornik, K. tseries: Time Series Analysis and Computational Finance. R package version 0.10–47., https://CRAN.R-project.org/package=tseries (2019).

[47] R Development Core Team. R: A Language and Environment for Statistical Computing, https://www.R-project.org/ (2018).

[48] Kendall, M. & Stuart, A. The advanced theory of statistics, Vol. 3, Griffin. pp. 410–414. (1983).

[49] Pohlert, T. trend: Non-Parametric Trend Tests and Change-Point Detection. R package version 1.1.1., https://CRAN.R-project.org/package=trend (2018).

[50] ESRI. ArcGIS Pro: Version 2.2.0. Redlands, CA: Environmental Systems Research Institute (2019).

[51] Carpenter TE (2001). Methods to investigate spatial and temporal clustering in veterinary epidemiology. Prev. Vet. Med..

[52] Kulldorff M (1997). A spatial scan statistic. Comm. Stat. Theory Methods..

[53] Kulldorff M, Nagarwalla N (1995). Spatial disease clusters: detection and inference. Stat. Med..

[54] Pfeiffer, D. U. *et al*. *Spatial Analysis in Epidemiology* (2008).

