# Author Correction: 50 Years of Cetacean Strandings Reveal a Concerning Rise in Chilean Patagonia

**DOI:** 10.1038/s41598-020-68367-7

**Published:** 2020-07-15

**Authors:** Mario Alvarado-Rybak, Frederick Toro, Joaquín Escobar-Dodero, Amy C. Kinsley, Maximiliano A. Sepúlveda, Juan Capella, Claudio Azat, Galaxia Cortés-Hinojosa, Natalia Zimin-Veselkoff, Fernando O. Mardones

**Affiliations:** 1 PhD Program in Conservation Medicine, Faculty of Life Sciences, Universidad Andres Bello, Republica 252, Santiago, Chile; 2 Sustainability Research Center (CIS), Faculty of Life Sciences, Universidad Andres Bello, Republica 252, Santiago, Chile; 3 Escuela de Medicina Veterinaria, Universidad Santo Tomás, Av. Limonares 190, Viña del Mar, Chile; 4 Department of Veterinary Population Medicine, College of Veterinary Medicine, University of Minnesota, Saint Paul, MN USA; 5 Chilean Patagonia Project, The Pew Charitable Trusts, Av. Costanera Andrés Bello 2233, Providencia, Santiago, Chile; 6 Whalesound Ltda, Lautaro Navarro, 1163 P2, Punta Arenas, Chile; 7 Department of Small Animal Clinical Sciences, College of Veterinary Medicine, Gainesville, University of Florida, Florida, 32610 USA; 8 School of Veterinary Medicine, Pontifical Catholic University of Chile, Marcoleta 391, 8330024 Santiago, Chile

Correction to: *Scientific Reports* https://doi.org/10.1038/s41598-020-66484-x, published online 11 June 2020

The original version of this Article contained an error in the number of Cetacean Stranding events recorded.

As a result of this, in the Abstract,

“We identified a total of 389 CS events affecting eight cetacean families, 21 genera, and 35 species, which represent more than 85% of the reported species richness for the country.”

now reads:

“We identified a total of 436 CS events affecting eight cetacean families, 21 genera, and 35 species, which represent more than 85% of the reported species richness for the country.”

Additionally, in the Results section under the subheading “Cetacean Stranding Events”,

“Between January 1968 and January 2020, a total of 441 CS events, affecting 1,607 stranded cetaceans, were recorded along the Chilean coast (Fig. 1). Most CS events (94.1%) were single (i.e., ≤ two individuals). There were also 18 mass strandings (three to 24 individuals, 4.1%) and nine unusually large mass stranding event (> 25 individuals, 2%). Cetacean strandings were reported every month; with 20.2% (n = 89) of total events occurring in January, 13.2% (n = 58) in February, and 8.4% (n = 37) in July. (Fig. 2). Spatially, at least one CS event was reported in 15 out of the 16 administrative regions of Chile. When the locations were aggregated by month, it became apparent that most events occurred in the regions of Valparaíso with 17% (n = 75), Magallanes with 14.5% (n = 64), and Coquimbo 11.3% (n = 50). On the opposite end, the region of Araucanía had the least number of reported events with only two strandings (0.5% of all events). The greatest number of CS events was observed in 2019 with 11.3% (n = 50), followed by the year 2018 with 10.7% (n = 47) and 2015 with 9.3% (n = 41). Regarding the numbers of stranded individuals, the greatest numbers of total stranded cetaceans were reported in March with 37.9% (n = 610), followed by July with 16.5% (n = 266) and April with 10.8% (n = 174) (Fig. 2). The Aysén and Magallanes regions accounted for most of the geographical distribution, with a 33.8% (n = 543) and a 31.5% (n = 506) of the total number of stranded individuals, followed by the Coquimbo region with 9.6% (n = 154). The year 2015 also accounted for the highest number of stranding individuals with 25.3% (n = 407) of the total, followed by the years 1989 and 2016 with 11.1% (n = 185) and 9.4% (n = 151) specimens. Overall, the median of stranded cetaceans in an stranding event was 2 with an interquartile range of 3; and the largest number of stranded cetaceans for a given event was 367 Sei whales that were reported at the Golfo de Penas area in the Magallanes region in March 2015^24^.”

now reads:

“Between January 1968 and January 2020, a total of 436 CS events, affecting 1,596 stranded cetaceans, were recorded along the Chilean coast (Fig. 1). Most CS events (94.1%) were single (i.e., ≤ two individuals). There were also 17 MSE (three to 24 individuals, 3.9%) and nine UME (> 25 individuals, 2.1%). Cetacean strandings were reported every month; with 20.4% (n = 89) of total events occurring in January, 13.3% (n = 58) in February, and 8.5% (n = 37) in July. (Fig. 2). Spatially, at least one CS event was reported in 15 out of the 16 administrative regions of Chile. When the locations were aggregated by month, it became apparent that most events occurred in the regions of Valparaíso with 17% (n = 74), Magallanes with 14.4% (n = 63), and Coquimbo 11.5% (n = 50). On the opposite end, the region of Araucanía had the least number of reported events with only two strandings (0.5% of all events). The greatest number of CS events was observed in 2019 with 11.2% (n = 49), followed by the year 2018 with 10.3% (n = 45) and 2015 with 9.4% (n = 41). Regarding the numbers of stranded individuals, the greatest numbers of total stranded cetaceans were reported in March with 38.2% (n = 610), followed by July with 16.6% (n = 266) and April with 10.9% (n = 174) (Fig. 2). The Aysén and Magallanes regions accounted for most of the geographical distribution, with a 34% (n = 543) and a 31.6% (n = 505) of the total number of stranded individuals, followed by the Coquimbo region with 9.6% (n = 154). The year 2015 also accounted for the highest number of stranding individuals with 25.5% (n = 407) of the total, followed by the years 1989 and 2016 with 11.5% (n = 185) and 9.4% (n = 151) specimens. Overall, the median of stranded cetaceans in an stranding event was 1 with an interquartile range of 1; and the largest number of stranded cetaceans for a given event was 367 Sei whales that were reported at the Golfo de Penas area in the Magallanes region in March 2015^24^.”

Also in the Results section, under the subheading “Cetacean species”,

“Cetacean stranding events were reported in eight cetacean families including 21 genera and 35 species. *Odontoceti* and *Mysticeti* species accounted for 74.8% (n = 330) and 25.2% (n = 111) of CS events, respectively; while odontocetes accounted for 66.8% (n = 1,073) and mysticetes for 33.2% (n = 534) of stranded cetaceans. Within stranded odontocetes, most events belonged to the *Delphinidae* 36.3% (n = 160), followed by the *Phocoenidae* 15.4% (n = 68) and *Physiteridae* 10.7% (n = 47) families. *Delphinidae* had the highest number of stranded individuals (n = 865), 80.6% of the *Odontoceti* order and 53.8% of all cetaceans. In the *Mysticeti* suborder, individuals of the *Balaenopteridae* family were the most frequently stranded with 96.3% (n = 514) of cases, followed by members of the *Balaenidae* family at 3.4% (n = 18). In term of stranding events, 87.4% (n = 97) were composed of *Balaenopterids*. If all events are considered together, the *Delphinidae* and *Balaenopteridae* families account for 58.3% (n = 257) of all stranding events, and for up to 85.8% (n = 1,379) of all cetaceans stranded through the years.”

now reads:

“Cetacean stranding events were reported in eight cetacean families including 21 genera and 35 species. *Odontoceti* and *Mysticeti* species accounted for 74.3% (n = 324) and 24.3% (n = 106) of CS events, respectively; while odontocetes accounted for 67.2% (n = 1,073) and mysticetes for 32.7% (n = 523) of stranded cetaceans. Within stranded odontocetes, most events belonged to the *Delphinidae* 36.3% (n = 160), followed by the *Phocoenidae* 20.9% (n = 68) and *Physiteridae* 14.5% (n = 47) families. *Delphinidae* had the highest number of stranded individuals (n = 865), 80.6% of the *Odontoceti* order and 53.8% of all cetaceans. In the *Mysticeti* suborder, individuals of the *Balaenopteridae* family were the most frequently stranded with 98.2% (n = 514) of cases, followed by members of the *Balaenidae* family at 1.3% (n = 7). In term of stranding events, 91.5% (n = 97) were composed of *Balaenopterids*. If all events are considered together, the *Delphinidae* and *Balaenopteridae* families account for 58.9% (n = 257) of all stranding events, and for up to 86.4% (n = 1,379) of all cetaceans stranded through the years.”

and,

“Only six odontocetes were classified as undetermined due to their advanced state of decomposition.”

now reads:

“Only six cetaceans were classified as undetermined due to their advanced state of decomposition.”

In the first row of Table 1 under the headings “N^o^ Individuals” and “N^o^ Events”,

“18” and “12”,

now read:

“7” and “7”

and under the heading “Common name”,

“Short-finned Whale” and “Long-finned Whale”

now read:

“Short-finned Pilot Whale” and “Long-Finned Pilot Whale”.

Figure 1 was incorrect in the original version of the Article (Fig. 1).

**Figure 1:**
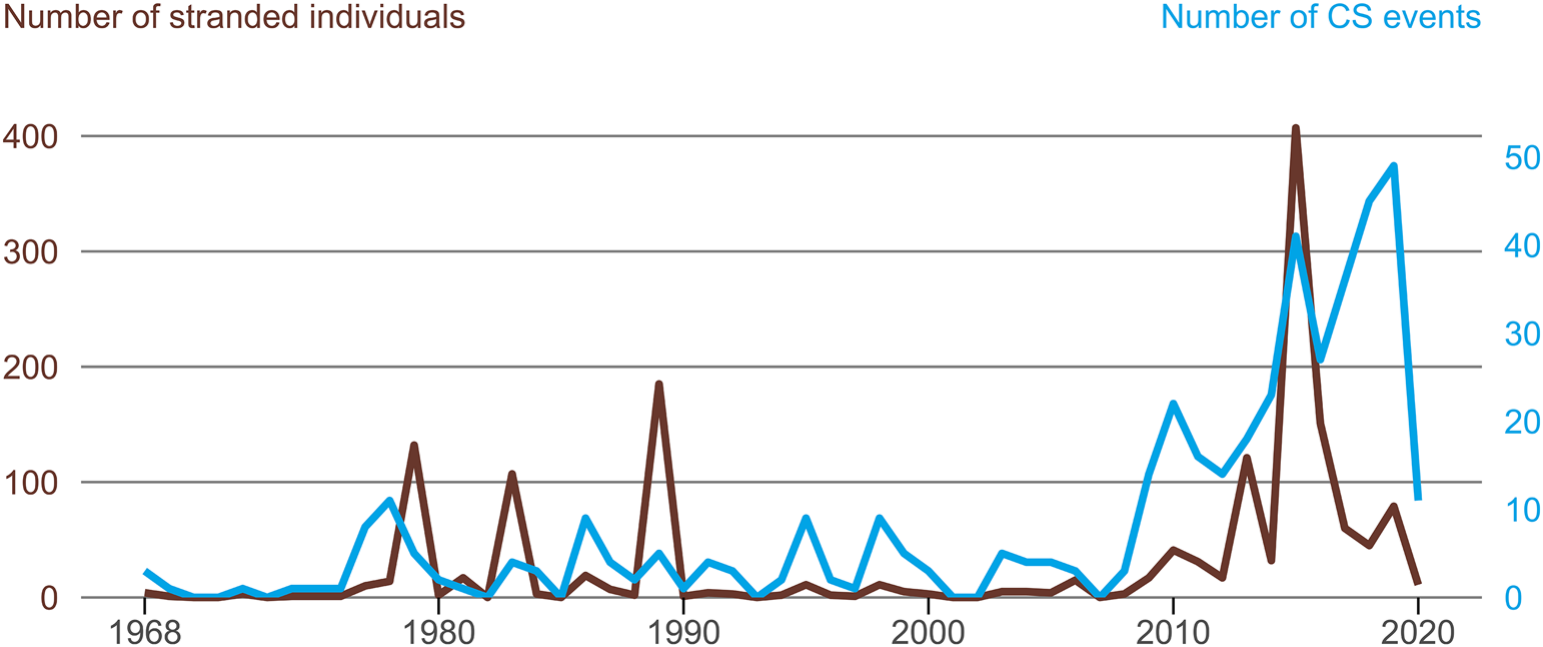
Observed number of stranded cetacean individuals (brown) and number of stranding events (blue line) from January 1968 to January 2020 in Chile.

Also as a result of the original error, in the Discussion section,

“We also discuss the within-year variation of reporting, the identification of hot spots in different areas of Chile and the species composition of the dataset. In the last year, there have been 61 CS events, 15 of them (24.6%) occurring between December 2019 and January 2020 (last summer season). All these CS events reported a total of 92 stranded individuals that were extensively distributed in the Chilean coast.”

now reads:

“We also discuss the within-year variation of reporting, the identification of hot spots in different areas of Chile and the species composition of the dataset. In the last year, there have been 60 CS events, 15 of them (25%) occurring between December 2019 and January 2020 (last summer season). All these CS events reported a total of 90 stranded individuals that were extensively distributed in the Chilean coast.”,

“In fact, from nine existent families, 24 genera and 41 species that have been reported in Chilean waters^26^, CS events reported here identified 88.9% of cetacean families, 87.5% and 85.4% of genera and species, respectively.”

now reads:

“In fact, from nine existent families, 24 genera and 41 species that have been reported in Chilean waters^26^, CS events reported here identified 88.9% of cetacean families, 87.5% and 85.3% of genera and species, respectively.”,

“This was observable for most species, but it was unusually high for *P. spinipinnis*, reported in 43 events. *Phocoena spinipinnis* are a group of porpoises that regularly strand in coastal areas worldwide^3^. It has been described that the leading causes of death are from bycatch and naval presence^28^. In our study, the strandings of *P. spinipinnis* took place for the most part in major ports and fisheries located in central Chile. A different situation occurred with B. borealis (n = 387), with 13 reported CS throughout the study period. However, a single event reported about 95% of total reported stranded individuals.

now reads:

“This was observable for most species, but it was unusually high for *P. spinipinnis*, reported in 66 events. *Phocoena spinipinnis* are a group of porpoises that regularly strand in coastal areas worldwide^3^. It has been described that the leading causes of death are from bycatch and naval presence^28^. In our study, the strandings of *P. spinipinnis* took place for the most part in major ports and fisheries located in central Chile. A different situation occurred with *B. borealis* (n = 414), with 17 reported CS throughout the study period. However, a single event reported about 88.6% of total reported stranded individuals.

and,

“The analysis of the within-year variation indicates that although CS events were reported at all times of the year, the months from February to April (summer and early autumn) and July (winter) account for 41% of all CS events.”

now reads:

“The analysis of the within-year variation indicates that although CS events were reported at all times of the year, the months from February to April (summer and early autumn) and July (winter) account for 41.9% of all CS events.”

Additionally, the original Article contained an error in the legend of Figure 3. As a result of this,

“(**a**) Stranding events (black dots) along Chile since 1968 to 2020. (**b**) Heatmap that illustrates density of stranding cases along Chile.”

now reads:

“(**a**) Cetacean stranding events (black dots) along Chile since 1968 to 2020. (**b**) Heatmap that illustrates density of stranding cases along Chile.”

Additionally, the original Article contained an error in the Author Contributions section, where,

“M.A.R. and F.T. led the project, designed the work, collected data and performed the systematic review. M.A.R., F.T., and F.O.M. wrote the paper, and J.E.D., M.S., B.T., C.S.A., J.C., G.C.H., N.Z.V. and A.C.K. helped to improve the manuscript. Time series, spatiotemporal analysis, visualization and plotting results were performed and described by M.A.R., J.E.D. and F.O.M.”

now reads:

“M.A.R. and F.T. led the project, designed the work, collected data and performed the systematic review. M.A.R., F.T., and F.O.M. wrote the paper, and J.E.D., M.S., C.A., J.C., G.C.H., N.Z.V. and A.C.K. helped to improve the manuscript. Time series, spatiotemporal analysis, visualization and plotting results were performed and described by M.A.R., J.E.D. and F.O.M.”

These errors have now been corrected in the HTML and PDF versions of this Article.

